# Negative optokinetic afternystagmus in larval zebrafish demonstrates set-point adaptation

**DOI:** 10.1038/s41598-019-55457-4

**Published:** 2019-12-13

**Authors:** Ting-Feng Lin, Mohammad Mohammadi, Ahmed M. Fathalla, Duygu Pul, Dennis Lüthi, Fausto Romano, Dominik Straumann, Kathleen E. Cullen, Maurice J. Chacron, Melody Ying-Yu Huang

**Affiliations:** 1 Department of Neurology, University Hospital Zurich, University of Zurich, Zurich, Switzerland; 2 Neuroscience Center Zurich (ZNZ), University of Zurich and ETH Zurich, Zurich, Switzerland; 3 Department of Biomedical Engineering, McGill University, Montreal, Quebec Canada; 4 Present Address: Monash Biomedicine Discovery Institute, Monash University, Melbourne, Australia; 5 Department of Biomedical Engineering, The Johns Hopkins University, Baltimore, Maryland USA; 6 Department of Physiology, McGill University, Montreal, Quebec Canada

**Keywords:** Oculomotor system, Reflexes

## Abstract

Motor learning is essential to maintain accurate behavioral responses. We used a larval zebrafish model to study ocular motor learning behaviors. During a sustained period of optokinetic stimulation in 5-day-old wild-type zebrafish larvae the slow-phase eye velocity decreased over time. Then interestingly, a long-lasting and robust negative optokinetic afternystagmus (OKAN) was evoked upon light extinction. The slow-phase velocity, the quick-phase frequency, and the decay time constant of the negative OKAN were dependent on the stimulus duration and the adaptation to the preceding optokinetic stimulation. Based on these results, we propose a sensory adaptation process during continued optokinetic stimulation, which, when the stimulus is removed, leads to a negative OKAN as the result of a changed retinal slip velocity set point, and thus, a sensorimotor memory. The pronounced negative OKAN in larval zebrafish not only provides a practical solution to the hitherto unsolved problems of observing negative OKAN, but also, and most importantly, can be readily applied as a powerful model for studying sensorimotor learning and memory in vertebrates.

## Introduction

Sensory-motor learning is essential for animals to ensure accurate performance and motor coordination during reflex and voluntary behaviors^1,2^. Because movement accuracy can be compromised by many factors, such as changes in the environment, injuries in the central or peripheral motor systems, and the inherent variability of central motor commands, the brain relies on mechanisms such as neural adaptation^3,4^ and habituation^5,6^ to better control movements. The optokinetic system, which produces reflexive eye tracking movements in response to full-field motion of the visual surround, provides advantageous means to study sensorimotor learning. This system is highly conserved across vertebrate species, and its main function is to stabilize the visual image on the retina, thereby allowing high-resolution vision^7,8^. The resultant optokinetic nystagmus (OKN, or the optokinetic response/reflex, OKR) eye movements are robust and stereotyped^7,9,10^.

The velocity storage mechanism is a multisensory element that “stores” the visual and head velocity information received from the optokinetic and vestibular systems, respectively^11,12^. Following the offset of visual stimulation, subjects continue to generate persistent eye movements in the dark – termed optokinetic afternystagmus (OKAN) – which are attributed to the velocity storage mechanism^11^. In human, other mammalian and non-mammalian species that were studied, the direction of the OKAN response is the same as the preceding OKN response and is termed positive OKAN^11,13–17^.

In addition to the positive OKAN, a negative OKAN (normally recorded after the cessation of positive OKAN), referring to afternystagmus beating in the opposite direction to the preceding OKN, has been identified in various organisms^16,18–23^. It is believed that the longer lasting negative OKAN is first masked by the shorter lasting positive OKAN; moreover, positive and negative OKAN seem to present a mutual waning and waxing relationship, as longer optokinetic stimulation leads to a weaker positive OKAN followed by stronger negative OKAN^19,24,25^. Furthermore, the manifestation of negative OKAN could be influenced not only by positive OKAN^20,26^, but also by smooth pursuit afternystagmus in foveated animals, in which the eyes continue moving in the same direction as the previous foveal tracking behavior^27–29^.

In the present study, we employed a larval zebrafish model to investigate negative OKAN. Taking advantage of the relatively inconspicuous positive OKAN and nonexistent smooth pursuit in the ocular motor system in larval zebrafish, robust negative OKAN could readily be elicited after sustained optokinetic stimulation in larvae at 5 days postfertilization (dpf). We observed the decay of OKN slow-phase velocity (SPV) during the optokinetic stimulation followed by negative OKAN with multiple time scales when the light was extinguished, or when images stopped moving. The SPV decay could be attributed to both sensory habituation and set-point adaptation of the retinal slip velocity; moreover, the set-point adaptation leads to the poststimulatory negative OKAN. This hypothesis is further supported by our mathematical simulations that fit the experimental data well when both mechanisms are incorporated. In summary, larval zebrafish offers a unique model to study sensorimotor learning by further investigations of the neurophysiological mechanisms that underlie negative OKAN.

## Results

### Negative OKAN in larval zebrafish

**Figure 1:**
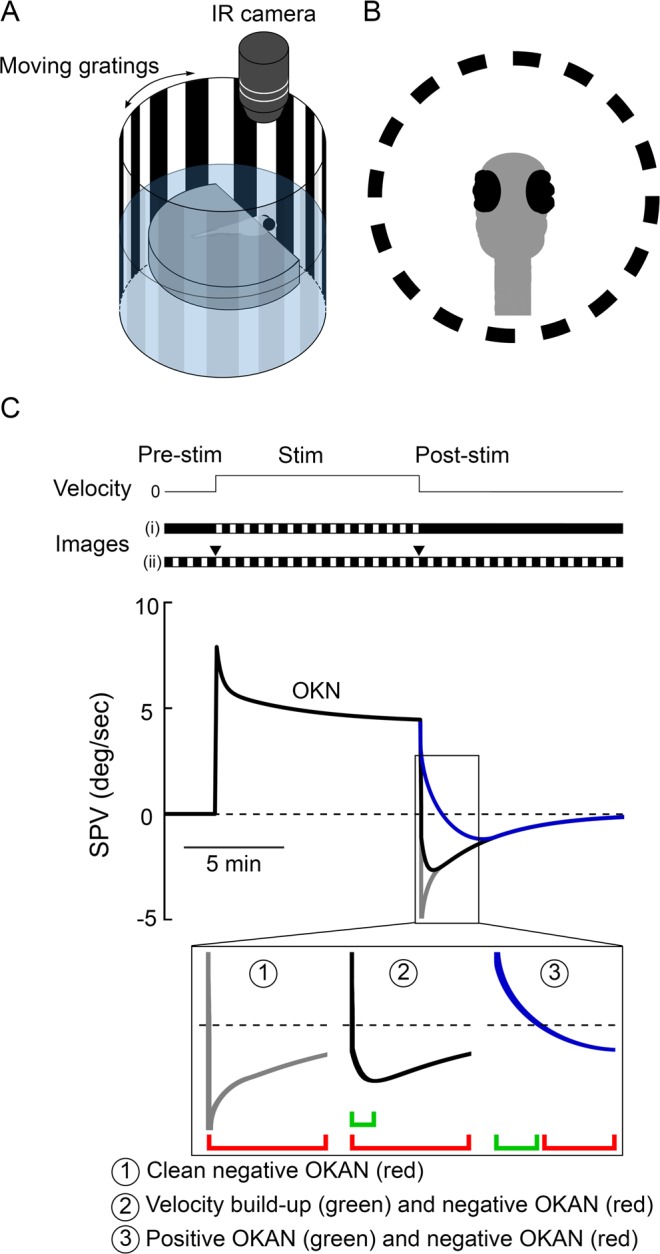
Experimental setup and visual stimulus condition. (**A**) Schematic drawing of the experimental setup. Individual five-day-old larvae were chosen randomly from clutches and individually fixed and tested. OKN was elicited by a computer-generated horizontally moving sinusoidal black and white vertical grating pattern projected onto a transparent screen wrapped around the cylinder. (**B**) Eye and body dislocation were recorded by an IR-sensitive camera, and the data were analyzed by custom-developed software written in MATLAB. (**C**) The visual stimulation protocol and a demonstrated SPV trace of OKN and OKAN. The horizontal black line on the top depicts the stimulus velocity. The two horizontal bars at the middle depict the stimulus images. Each recording started with 5 min in the dark (i) or with a stationary grating around the fish (ii) as the prestimulatory phase, followed by an OKN stimulation of different durations as the stimulatory phase, and concluded with another dark (i) or stationary grating period (ii) as the poststimulatory phase. The two triangles above (ii) depict the transitions between moving and stationary grating periods as previously described. The bottom plot shows SPV traces of OKN followed by negative OKAN (red) affected by different levels of velocity-storage components. ① A hypothetical clean negative OKAN without the influence of velocity storage mechanism. ② A negative OKAN with a velocity build-up phase (green) at the transition, attributing to the velocity storage mechanism. ③ A negative OKAN first masked by the shorter lasting positive OKAN (green).

**Figure 2:**
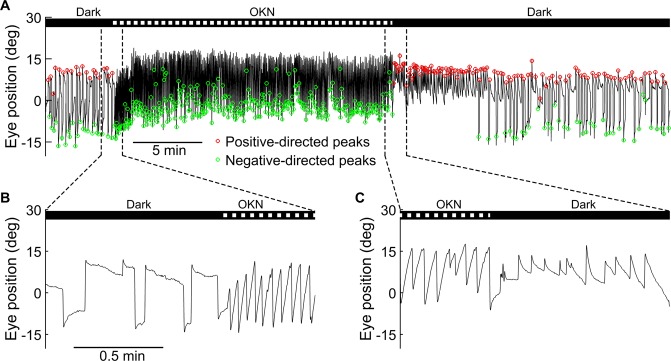
Negative OKAN of a zebrafish larva. Visual stimuli over time: 0–5 min, dark; 5–25 min, vertical gratings rotating horizontally at a constant velocity of 10 deg/sec in one direction; 25–45 min, dark period. (**A**) Typical eye-position trace of a larva in the dark, during the 20-min optokinetic stimulation, and during negative OKAN in the dark. Positive and negative quick-phase velocity peaks are marked in red and green, respectively. (**B**,**C**) Magnifications of **A** to demonstrate the transition phase from prestimulation to OKN and from OKN to negative OKAN, respectively.

To test set-point adaptation in the ocular motor system by observing OKN and the resulting OKAN under different stimulus conditions, we recorded the eye movements of the larval zebrafish during 3 phases (see Methods; Fig. 1): First a 5 min base line period in the dark, next followed by 20 min of unidirectional 10 deg/sec optokinetic stimulation and finally another 20 min of darkness. Representative eye-position traces are shown in Fig. 2. We identified quick phase eye movements in both positive and negative directions (see Methods) for recorded eye movement (Fig. 2A). Magnification of the transitions between each of the three phases in Fig. 2A are shown in Fig. 2B (prestimulation to stimulation) and Fig. 2C (stimulation to poststimulation). In the dark before the optokinetic stimulation, eye movements consisted of bidirectional spontaneous saccades followed by centripetal slow eye drifts (Fig. 2B). After sustained optokinetic stimulation, we observed the negative OKAN for a duration of approximately 7 min (Fig. 2A,C). Interestingly, during negative OKAN, the beating field deviated toward the side of the previous stimulus direction (0 to 15 deg, Fig. 2A). In contrast, the eyes moved across a broader range during spontaneous eye movement in both the pre- and poststimulatory phases (±15 deg, Fig. 2A). Moreover, we could record a SPV accelerating phase (build-up phase, Supplementary Fig. S1A and S1B) or a “silent” period of more than 30 seconds (Supplementary Fig. S1C) during the initial period of negative OKAN, suggesting the velocity storage of the preceding OKN.

### Quantification of negative OKAN in larval zebrafish

**Figure 3:**
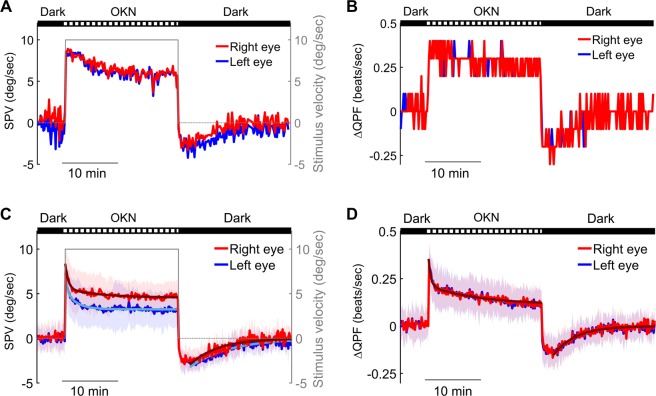
SPV and ∆QPF of negative OKAN. (**A**,**C**) The average SPV of every 10 sec during the entire 45-min recording period (5 min in the dark, 20 min of optokinetic stimulation at a constant velocity of 10 deg/sec in one direction, and another 20 min in the dark). (**A**) Shows the single subject data of Fig. 2, and (**C**) shows the average values of 15 subjects. The gray line depicts the stimulus velocity with a second y-axis on the right side. (**B**,**D**) The ∆QPF of every 10 sec during the entire recording as described above. (**B**) Shows the single subject data of Fig. 2, and (**D**) shows the average values of 15 subjects. The data collected from the right eye and left eye are shown as red and blue lines, respectively. Cyan and dark red lines are the fitting curves of the OKN adaptation and negative OKAN decay in the dark. The first 2 min of the OKAN velocity data were excluded from the fitting to minimize the masking effect of the velocity-storage components during the build-up phase of the absolute OKAN velocity. Shaded areas indicate standard deviation.

To quantify negative OKAN, we analyzed SPVs between each quick phase. The average SPVs of every 10 sec over the entire 45-min recording period are shown in Fig. 3A (the single subject data of Fig. 2) and 3C (the average values of 15 subjects). In response to counterclockwise-rotating optokinetic stimulation (which was applied throughout the study), right eyes displayed faster SPV than left eyes due to the temporal-to-nasal asymmetry in lateral-eyed animals^7,16,30–41^. We additionally computed the quick-phase frequency (QPF) and compared the pre- and poststimulatory values. We categorized the quick phases into positive and negative groups (Fig. 2A) and subsequently estimated the frequency of each group in a time window of 10 sec. The ∆QPF was calculated by subtracting the positive QPF from the negative QPF. The ∆QPFs are shown in Fig. 3B (the single subject data of Fig. 2) and 3D (the average values of 15 subjects). We calculated Pearson correlation between the average SPVs and the corresponding ∆QPFs for the same 10-sec time window. Our data showed a significant correlation between SPV and ∆QPF in larval zebrafish (Supplementary Fig. S2; Supplementary Table S1), similar results were found previously in human and mouse subjects^42,43^. Thus, we suggest that both SPV and QPF analyses give comparable estimates of the negative OKAN.

### Time course of negative OKAN in larval zebrafish

We estimated the dynamics of OKN adaptation by fitting the decline of the SPV during the optokinetic stimulation phase with a second-order exponential decay model (Fig. 3C; OKN adaptation time constants ±95% confidence interval: 0.35 ± 0.36 min and 1.93 ± 0.82 min (left eye); 0.54 ± 0.23 min and 6.15 ± 4.66 min (right eye)). Using a similar method, we also estimated the decay of negative OKAN by fitting the poststimulatory SPV with a first-order exponential decay model (Fig. 3C: OKAN decay time constant ±95% confidence interval: 7.03 ± 0.79 min (left eye) and 5.02 ± 0.81 min (right eye)). Note, to avoid the velocity build-up (accelerating) phase (see ② in Fig. 1C), the first 2 min of the OKAN velocity data were excluded from the fitting. We also estimated the decay time courses of ∆QPF in the same way as with the SPV data: second-order exponential decay fitting for OKN adaptation and first-order exponential decay fitting for negative OKAN (Fig. 3D; OKN adaptation time constant ±95% confidence interval: 0.41 ± 0.21 min and 8.86 ± 1.28 min (left eye); 0.39 ± 0.17 min and 8.68 ± 1.28 min (right eye); OKAN decay time constant ±95% confidence interval: 4.22 ± 0.60 min (left eye) and 3.94 ± 0.55 min (right eye)).

### The dependence of negative OKAN on optokinetic stimulus duration

**Figure 4:**
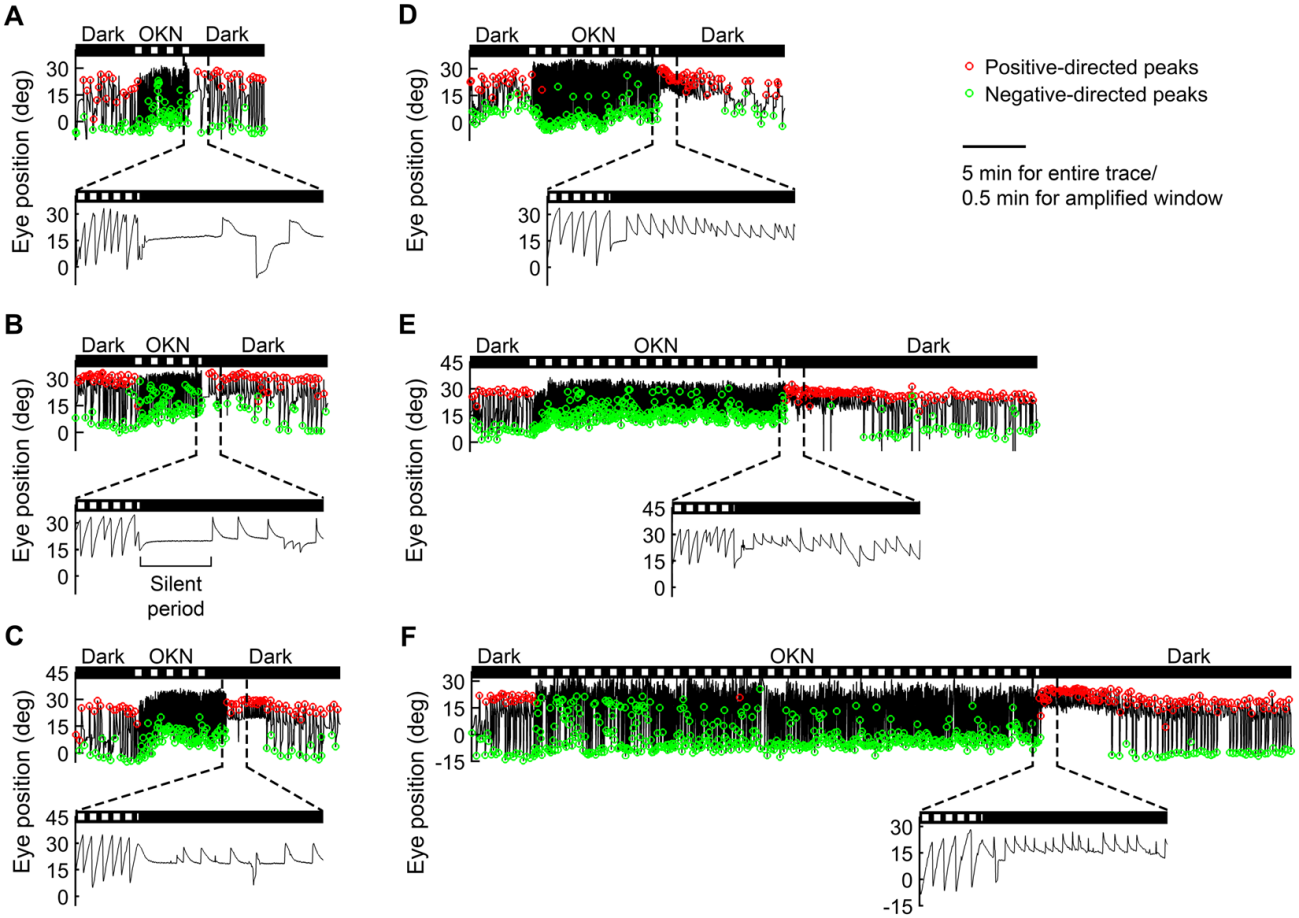
Negative OKAN recorded after different optokinetic stimulation durations. Typical eye-position traces of individual zebrafish larvae recorded in 5 min of darkness, followed by different time periods of optokinetic stimulation (**A** to **F**: 4, 5, 7, 10, 20 and 40 min) and then different durations of darkness (**A** to **F**: 5, 10, 10, 10, 20 and 20 min). The stimulus velocity was 10 deg/sec. Positive and negative quick-phase velocity peaks are marked in red and green, respectively. Magnifications demonstrate the transition phases from OKN to darkness.

**Figure 5:**
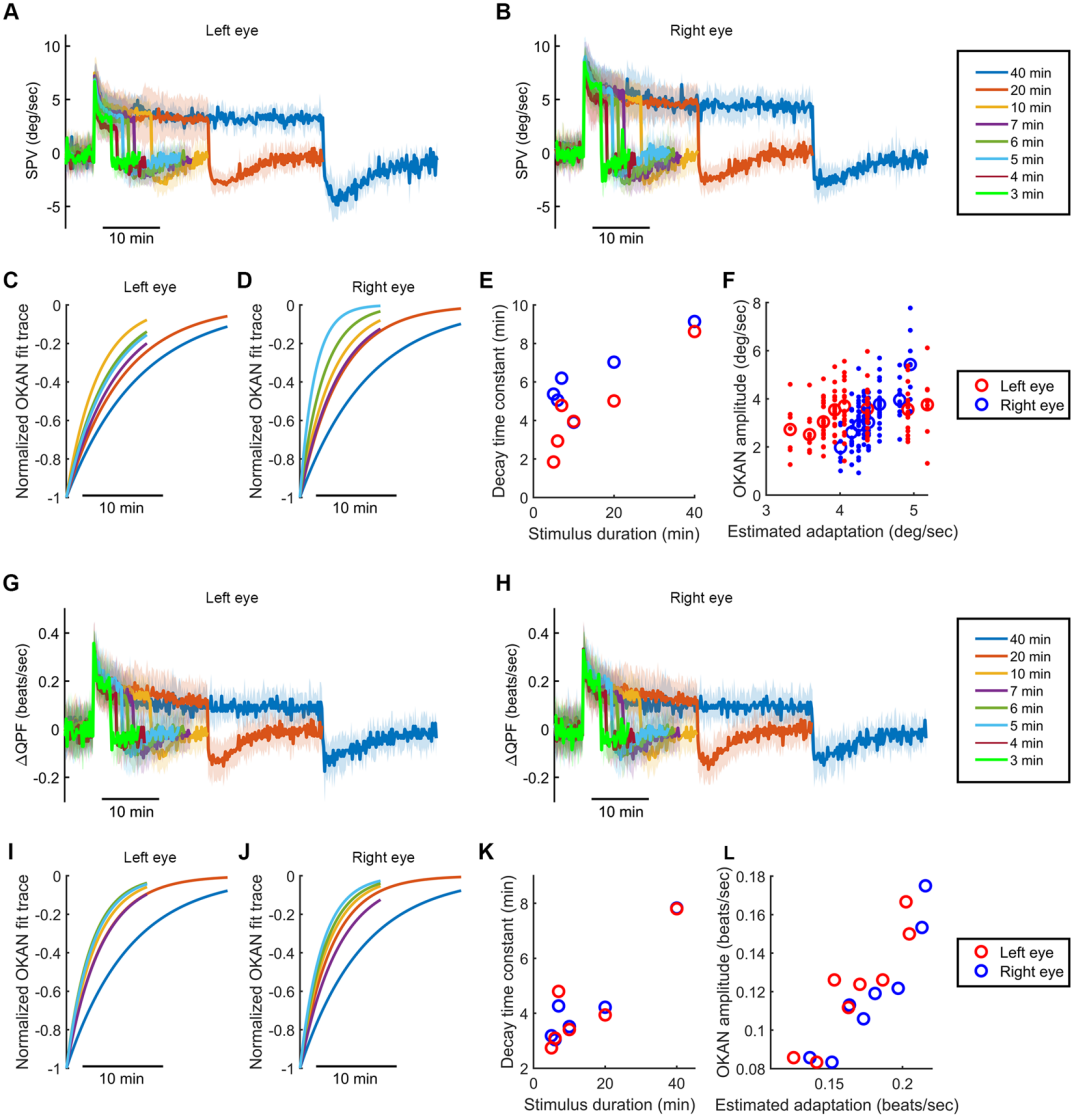
Negative OKAN dynamics in relation to stimulus duration at a constant velocity of 10 deg/sec in one direction. (**A**,**B**) and (**G**,**H**) Mean ± standard deviation of the SPV (**A,B**) and ∆QPF (**G,H**) in every 10 sec during the entire recording with different stimulus durations (5 min in the dark, 3, 4, 5, 6, 7, 10, 20, and 40 min of optokinetic stimulation at a constant velocity of 10 deg/sec in one direction, and 5, 6, 10, 10, 10, 10, 20, and 20 min in the dark, respectively; n = 7, 12, 23, 17, 21, 23, 15, and 8). (**C,D**) and (**I**,**J**) Normalized fitting curves of negative OKAN decay after 5, 6, 7, 10, 20, and 40 min of optokinetic stimulation estimated from the SPV (**C,D**) and ∆QPF (**I,J**). (**E** and **K**) The decay time constants estimated from the SPV (**E**) and ∆QPF (**K**) were plotted against stimulus duration. (**F** and **L**) Negative OKAN amplitudes after 3, 4, 5, 6, 7, 10, 20, and 40 min of optokinetic stimulation estimated from the SPV (**F**) and ∆QPF (**L**) were plotted against estimated OKN adaptation. Blue circles indicate left-eye data, and red circles indicate right-eye data.

To further study and estimate the negative OKAN dynamics, we subjected zebrafish larvae to optokinetic stimulation of different durations. First, we show representative eye traces after 4, 5, 7, 10, 20 and 40 min of 10 deg/sec optokinetic stimulation (Fig. 4). A robust negative OKAN, defined as continuous beating in the same direction as the previous stimulus, lasted for a longer duration following a longer optokinetic stimulation. The magnified traces show the transition phases between stimulatory and poststimulatory periods, in which one can clearly visualize the more sharpened (i.e., faster) poststimulatory eye movement and the increased poststimulatory beating frequency as the stimulus duration is prolonged. The average SPV and ∆QPF following 3, 4, 5, 6, 7, 10, 20 and 40 min of stimulation are shown in Fig. 5A,B,G,H, respectively. To better estimate the time span of negative OKAN following different stimulus durations, we subjected the SPV and ∆QPF of negative OKAN to exponential decay fitting. Due to the relatively low negative OKAN amplitude that largely decreased the signal-to-noise ratio, 3- and 4-min data were not subjected to fitting. The SPV decay fit traces were normalized to the peak and were plotted in Fig. 5C (left eye) and 5D (right eye), and the estimated time constants were plotted in Fig. 5E (Supplementary Table S2). The SPV data showed a significant correlation between stimulus duration and the decay time constant in both eyes (r = 0.87, p = 0.023, n = 6 in left eye and r = 0.93, p = 0.007, n = 6 in right eye, Pearson correlation analysis). The normalized fit traces of ∆QPF decay are shown in Fig. 5I,J, and the estimated time constants are shown in Fig. 5K (Supplementary Table S2). The ∆QPF data also showed a significant correlation between negative OKAN decay time course and stimulus duration (r = 0.95, p = 0.004, n = 6 in left eye and r = 0.89, p = 0.017, n = 6 in right eye, Pearson correlation analysis).

### Correlation between the amplitude of the negative OKAN and the adaptation extent to the preceding optokinetic stimulus

To study the correlation between the ultimate OKN adaptation, which is defined as the maximal reduction of OKN SPV or ∆QPF from the initial peak values to the close-to-plateau phase values during stimulation (estimated by fitting with a second-order exponential decay function), and the negative OKAN amplitude, which is defined as the absolute peak velocity or peak ∆QPF of OKAN (Fig. 5A,B,G,H), we performed a series of Pearson correlation analyses. The average OKAN amplitudes were plotted over the estimated OKN adaptation (Fig. 5F,L). The velocity data collected from both eyes showed significant correlations between estimated OKN adaptation and average negative OKAN amplitude (Fig. 5F; r = 0.96, p = 2.095e-04, n = 8 (left eye) and r = 0.77, p = 0.026, n = 8 (right eye), Pearson correlation analysis). Similarly, the ∆QPF data collected from both eyes also (Fig. 5L) showed a significant correlation between estimated OKN adaptation and average negative OKAN amplitude (r = 0.93, p = 7.295e-04, n = 8 (left eye) and r = 0.92, p = 0.001, n = 8 (right eye), Pearson correlation analysis). In summary, the OKN adaptation and the negative OKAN displayed a corresponding increase in their size, which may suggest shared underlying mechanisms of both phenomena.

### Altered eye drift properties during negative OKAN in poststimulatory darkness

**Figure 6:**
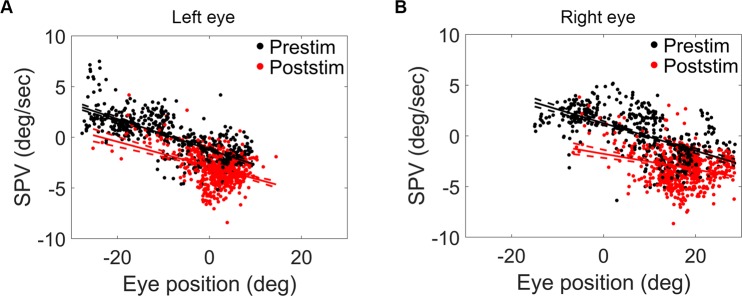
Velocity-position relationship of left eye (**A**) and right eye (**B**). Median velocity against median eye position in the first second of slow phases were plotted. Black dots are data collected during 5-min prestimulatory darkness and red dots during the first 5 min of poststimulatory darkness. Regression lines of prestimulatory and poststimulatory data were drawn with 95% confidence interval (dash line).

In animals, velocity-to-position integrator receives velocity signal and generates position signal to hold the gaze^8^. When the integrator is leaky (i.e. when the eyes cannot be held steady at an eccentric position), position signal decays exponentially, and thus the eye drifts back to the central position in the dark. Due to the leaky velocity-to-position neural integrator in larval zebrafish^44^, eye beatings toward more eccentric positions during negative OKAN could lead to a faster centripetal eye drifts and therefore an increase in the measured SPV. During both OKN and negative OKAN periods, eye beating fields deviated toward the side of the optokinetic stimulus direction (−5 deg to + 15 deg for OKN and 0–15 for OKAN, Fig. 2A), raising the concern of the possibility that the observed negative OKAN (or the increased poststimulatory SPV in darkness) could be simply leaky integrator-resulting centripetal eye drifts at more eccentric positions following asymmetric eye beatings. Thus, we drew the regression lines of the median velocity on median position of each slow phase in pre- and poststimulatory darkness. The result revealed a left/downward shift of the regression line after 20 min of stimulation in both eyes (Fig. 6), suggesting changed position-to-SPV relationship. Besides SPV, we also analyzed changes of the null position of the eye: Null positions are estimated as the x-intercept of regression lines, which are shifted from −8.02 deg to −23.56 deg in left eye and from 9.19 deg to −22.81 deg in right eye (calculated from the estimates listed in Supplementary Table S3). We additionally estimated the null position by using the entire slow phase data for the velocity-position plot which gave a similar result (data not shown).

**Figure 7:**
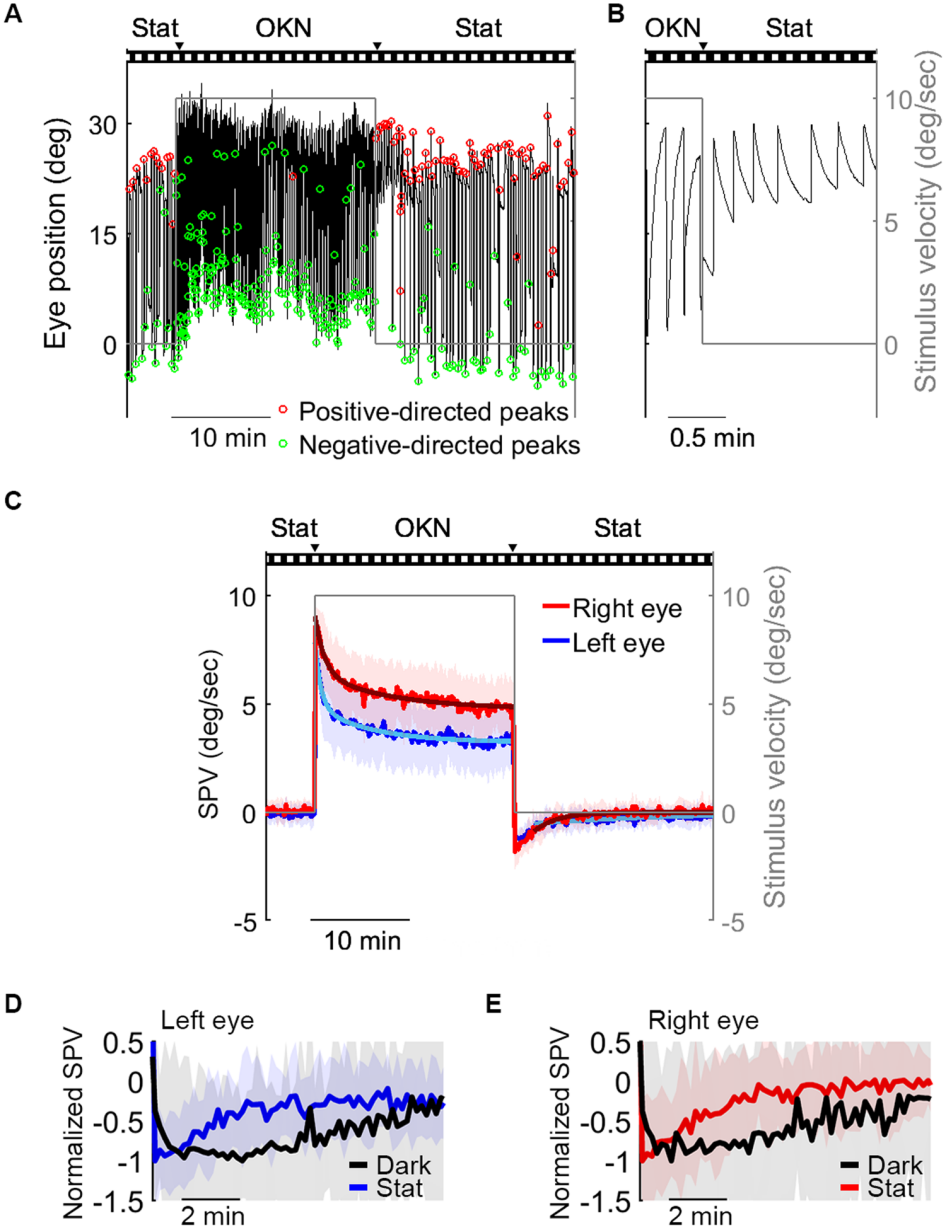
Negative OKAN of a zebrafish larva presented with the stationary gratings. Visual stimuli consisting of vertical gratings were presented in three phases: 0–5 min, 0 deg/sec; 5–25 min, 10 deg/sec in one direction; 25–45 min, 0 deg/sec. (**A**) Typical eye-position trace of a larva in the stationary phase, during the 20-min optokinetic stimulation, and during the negative OKAN with stationary gratings. Positive and negative quick-phase velocity peaks are marked in red and green, respectively. (**B**) Magnifications of **A** to demonstrate the transition phase from OKN to negative OKAN. (**C**) Mean ± standard deviation (n = 25) of the SPV every 10 sec during the entire 45-min recording period. The gray line depicts the stimulus velocity with a second y-axis on the right side. The triangles above the visual stimulation protocol depict the transitions between moving and stationary grating periods as previously described (Fig. 1). (**D**,**E**) OKAN SPV normalized by the peak value of left eyes (**D**) and right eyes (**E**) under stationary gratings (blue or red line) and in darkness (black line) during the first 10 min.

We also tested the negative OKAN under stationary gratings because the influence of the leaky velocity-to-position neural integrator on poststimulatory SPV should be minimized in this viewing condition as the optokinetic system would normally stabilize the gaze (see Fig. 2A by Chen *et al*.^44^). In our data, eye position traces revealed clear negative OKAN under stationary gratings (Fig. 7A,B); moreover, the SPV under stationary gratings was faster after optokinetic stimulation compared to the baseline value before optokinetic stimulation (Fig. 7C). After exclusion of the first 2-min post-optokinetic stimulation data, the estimated negative OKAN decay time constants ± 95% confidence intervals are 13.55 ± 2.91 (left eye) and 2.25 ± 0.49 min (right eye), respectively. This fitting period selection is identical to that of the exponential fit of poststimulatory SPVs for minimizing the influence of velocity build-up phase (Fig. 4). However, since in this case there was no observable speed build-up phase at the beginning of the negative OKAN (Fig. 7D,E), we performed another time constant estimation by fitting the negative OKAN SPV data of the entire postoptokinetic stationary period and obtained time constants ± 95% confidence interval of 7.28 ± 0.96 min (left eye) and 2.41 ± 0.23 min (right eye), respectively.

### Conceptual and mathematical model of negative OKAN

Based on the empirical data of OKN and negative OKAN in larval zebrafish, we hypothesize that sustained optokinetic stimulation leads to a set-point adaptation of the retinal slip velocity, which is reflected by the decreased SPV of OKN during the stimulation and manifested as negative OKAN in poststimulatory darkness (e.g., Figs. 3A,C, 5A,B). However, the SPV reduction cannot be explained by the set-point adaptation alone since the “adapted” amount (i.e. SPV reduction during OKN) is larger than the negative OKAN amplitude (e.g., Figs. 3A,C, 5A,B). Thus, we further suggest sensory habituation as another underlying mechanism that takes place simultaneously during the sustained stimulatory period.

**Figure 8:**
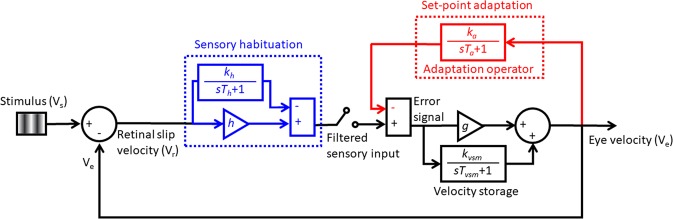
Conceptual and mathematical model of negative OKAN. The black part represents the optokinetic negative feedback control of the retinal slip velocity incorporating the velocity storage mechanism. The red part describes the set-point adaptation. Adaptation operator is composed of a leaky velocity integrator. *T*_*a*_ is time constant, while the reciprocal of *k*_*a*_ indicates how leaky the integrator is. The blue part describes the sensory habituation, which is composed of one gain and one leaky integrator. Sensory habituation adjusts how efficiently the initial sensory input can be converted into electrochemical signals. *T*_*h*_ is time constant, while the reciprocal of *k*_*h*_ indicates how leaky the integrator is.

To test these proposals, we simulated the conceptual model depicted in Fig. 8. The control system sends a motor command to the eyes in response to an error signal, which is the difference between the retinal slip velocity and its “internal” set point. The set point of the retinal slip velocity is normally 0 because the purpose of the optokinetic system is to stabilize the image on the retinae. The motor command simultaneously charges the adaptation operator (i.e. a leaky velocity integrator, the red component in Fig. 8) that defines the set point; therefore, over a sustained period of optokinetic stimulation, the change of the set point allows the optokinetic system to slow down its eye tracking without increasing the error signal despite of an increase in actual retinal slip. The habituation transfer function serves as a sensory filter that reduces the sensory input (i.e. the actual retinal slip velocity) at time. Once the light is extinguished, the current set point is subtracted from the sensory input of 0 (i.e. no visual input in the dark), which leads to an error signal of the set-point value but with an opposite sign. This error signal then drives the eyes to move in the opposite direction, i.e. the negative OKAN. The decay of negative OKAN represents the discharge of the set-point adaptation operator.

**Figure 9:**
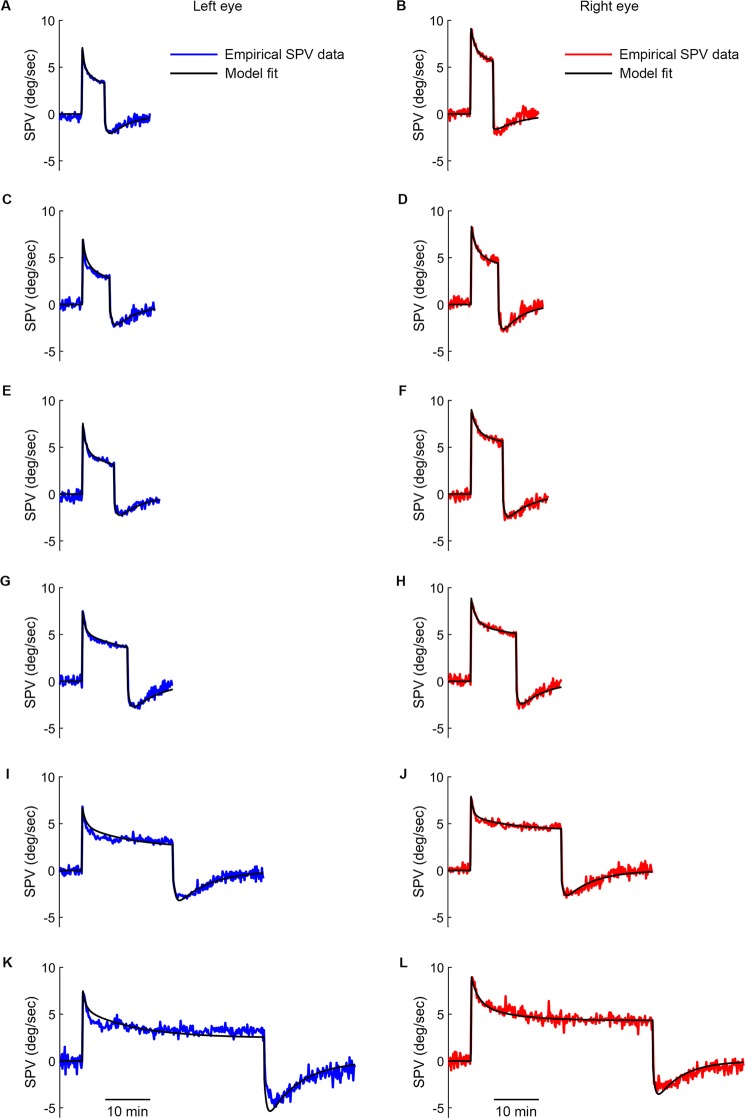
Simulated results compared to average SPV with different stimulus durations. Simulated SPVs (black lines) under 5- (**A-B**), 6- (**C-D**), 7- (**E-F**), 10- (**G-H**), 20-min (**I-J**) and 40-min (**K-L**) optokinetic stimulation of 10 deg/sec. The data collected from the left eye and right eye are shown as blue and red lines, respectively (see Fig. 5A,B). The optimized parameters are shown in Supplementary Table S4.

Figure 9 demonstrates the model simulations and the empirical SPVs. Supplementary Table S4 lists all estimated parameters and goodness of fit. In addition, we also show the simulation result of including either only the set-point adaptation transfer function or the habituation transfer function to further demonstrate the necessity of having both transfer functions in the model to explain our experimental data (Supplementary Fig. S3 and Supplementary Table S5). Notably, when we only include sensory habituation transfer function (Supplementary Fig. S3A), the model readout shows no negative OKAN (Supplementary Fig. S3B). Furthermore, when we only include set-point adaptation transfer function (Supplementary Fig. S3C), the simulation only fits the negative OKAN part (Supplementary Fig. S3D). Thus, computer simulation leads to the same conclusion as the empirical data that negative OKAN is the result of set-point adaptation; however, without sensory habituation we could not explain the dynamical changes of SPVs during a sustained period of optokinetic stimulation.

## Discussion

In this present study, we observed that the OKN responses of zebrafish show velocity adaptation in response to sustained visual stimulation, which, following the offset of the visual stimulus, is manifested as negative OKAN with shifted null position. We proposed a mechanism comprising multiple time course set-point adaptation of retinal slip velocity and using mathematical modeling we confirm that simulation of such a mechanism provides an excellent match to our empirical data.

### Larval zebrafish as a novel model to study negative OKAN

The evolutionary difference in ocular motor circuits among animals and the variation in experimental paradigms might be two of the most important factors determining the various dynamics of negative OKAN documented in the literature. For instance, the smooth pursuit afternystagmus^27,28^, which occurs after foveal tracking of a moving target, likely masks or overshadows the negative OKAN in humans^29^. Afoveate animals are usually believed to display no robust smooth pursuit^45,46^. However, with only visual streaks, rabbits can display afternystagmus in the preceding OKN direction in darkness for more than 50 min^25^. This time scale is more similar to smooth pursuit afternystagmus than to positive OKAN, which is believed to last up to two minutes. Taking advantage of the fact that larval zebrafish perform no smooth pursuit and only inconspicuous positive OKAN, we were able to study the dynamics of negative OKAN independently. Our results are consistent with a recent scientific report on set-point adaptation in the vestibular system which suggests motor learning takes place on multiple time scales in an adaptive process^47^.

### Velocity storage mechanism and negative OKAN in larval zebrafish

For a very long time, the optokinetic and the vestibular systems have been believed to share the same velocity-storage element^48^, which is fed by both visual and vestibular signals and is modified by gravity^12^. Positive OKAN reveals the discharge of the velocity storage^11^. In humans, labyrinth defects markedly diminishes positive OKAN^18,49,50^; therefore, vestibular input has been considered crucial for positive OKAN generation. The underdeveloped vestibular apparatus in larval zebrafish^51^ has been proposed to account for the missing positive OKAN in the animal^10^. Chen and colleagues proposed that the velocity-to-position neural integrator in larval zebrafish is not fully developed and leaky so that the velocity input from velocity storage mechanism fades away quickly, hence the difficulty in observing the positive OKAN^44^. Our data in the present study suggested a similar idea based on different pieces of evidence: we observed a quiet period (Supplementary Fig. S1C and Fig. 4B) or a build-up phase of the absolute SPV of negative OKAN for no more than 2 min (Supplementary Fig. S1A and S1B, and Fig. 7D,E). Thus, our results suggest that despite the fact that a positive OKAN is lacking, negative OKAN is initially suppressed, most likely by velocity-storage components, further supporting velocity storage mechanism being at least partially developed and functional in 5-dpf zebrafish larvae.

Interestingly, under the stationary grating condition such a negative OKAN build-up phase has not been observed (Fig. 7D,E). Waespe and colleagues reported that a 5-sec stationary grating image exposure immediately after an optokinetic stimulation shortened the positive OKAN duration and increased both the duration and velocity of negative OKAN in primates^26^. Our data in larval zebrafish are consistent with their finding and suggest that while negative OKAN continues for minutes under the exposure of a stationary image surround, the velocity storage is discharged right away (Fig. 7). To adapt quickly to the dynamical world, it has been proposed that the cerebellum is sensitive to variation in visual and vestibular signals and erases the previously developed velocity storage^52^.

### Negative OKAN demonstrates a set-point adaptation in the optokinetic system

This is the first time that set-point adaptation of retinal slip velocities has been applied to a model for negative OKAN. Previously, Leigh *et al*. suggested that the occurrence of negative OKAN is attributable to an adaptive mechanism^53^, a notion that is similar to the set-point adaptation. However, one important phenomenon during optokinetic set-point adaptation, the decrease in SPV during sustained OKN, has not been discussed in the past with the exception of a recent report in zebrafish larvae^54^. We speculate that, despite of the underlying set-point adaptation, the decreased SPV has not been observed in other rodent or primate models due to the prototypical cerebellar learning behavior, which on the contrary would increase the SPV gain during sustained optokinetic stimulation^55,56^. Without an observation of a decreased OKN gain, one is not able to introduce the set-point adaptation to the negative OKAN model.

In contrast, zebrafish larvae do not appear to show such a gain-increase adaptation during optokinetic stimulation (unpublished data) and therefore can serve as a suitable model to test the set-point adaptation hypothesis for the negative OKAN. Our present study provides clear evidence for a decline in SPV during sustained OKN, indicating the underlying mechanism of set-point adaptation. However, set-point adaptation alone cannot explain the difference between the reduced SPV during OKN and the negative OKAN amplitude in the empirical data. Our simulations with only a set-point adaptation operator, underestimated adaptation (Supplementary Fig. S3D), while the addition of sensory habituation to our model provided dynamics that better fit our data (Fig. 8). Indeed, retinal ganglion cells (RGCs) in larval zebrafish^54^ demonstrate sensory adaption (attenuated response over time) to visual stimuli. Thus we speculate that sustained optokinetic stimulation leads to a filtered (sensory adapted) input at the first stages of visual processing (e.g. retina) before the signal is transmitted further downstream where set-point adaptation then occurs. Taken together, both our empirical data and the model simulation results suggest that the combined effects of sensory habituation and set-point adaptation and reduce the SPV of OKN. Set-point adaptation alone, however, contributes to the negative OKAN. It remains to be determined exactly how these two mechanisms are implemented at the circuit level.

## Methods

All experiments in this study were carried out in agreement with the Federal Veterinary Office of Switzerland (FVO) guidelines for animal welfare and were run and designed in accordance with the Association for Research in Vision and Ophthalmology (ARVO) Statement for the Testing of Animals in Ophthalmic and Vision Research. Specially, no ethical approval is necessary for studies on larvae before they start independent feeding (120 hpf/5dpf), which is in accordance with the TSchV art. 112 stating experiments involving larvae that already ingest food freely need an approved license, which does not apply to this study.

### Fish breeding and upkeep

AB and TU wild-type zebrafish lines were bred and maintained in 28 °C E3 (5 mM NaCl, 0.17 mM KCl, 0.33 mM CaCl_2_, and 0.33 mM MgSO_4_) as described previously^57,58^ under a cycle of 14 hours of light/10 hours of darkness. Larvae at the age of 5 dpf were used for the experiments.

### Experimental overview

The zebrafish larvae were placed in the center of an optokinetic cylinder where we could surround the fish with moving or stationary visual stimuli (Fig. 1A,B). The experiments consisted of three phases: First, a baseline period in which spontaneous eye movements were recorded either in darkness or in the presence of illuminated stationary gratings; next, an optokinetic stimulatory phase (OKN); and finally, a postoptokinetic-stimulatory phase (OKAN) in darkness or surrounded by illuminated stationary gratings. These three phases are shown in the schematic diagram in Fig. 1C, where hypothetical negative OKANs are affected by different levels of velocity-storage components (Fig. 1C, ① to ③ show the influence of weak to strong velocity-storage components). Eye positions were recorded during all three phases. The median of the SPV was computed for further analysis of OKN and OKAN (the bottom of Fig. C).

### Stimulation of optokinetic nystagmus

All experiments were performed between 8:00 AM and 7:00 PM. Zebrafish larvae were restrained in low-melting agarose and placed in the center of an optokinetic drum without immobilizing the eyes. OKN was evoked through the usage of four digital light projectors (Samsung SP-H03 Pico Projector). The optokinetic stimulus consisted of a moving, computer-controlled black and white vertical sinusoid grating pattern with 100% contrast (maximum illumination 1524 lux) and spatial frequency of 0.053 cycles per degree projected onto a transparent screen at an angular velocity of 10 deg/sec, with stimuli lasting 3, 4, 5, 6, 7, 10, 20, 30 or 40 min. A custom-made program written in LabVIEW (National Instruments, Austin, Texas, USA)^44^ was used to control all the experimental processes and conditions such as frame processing, data recording, visual stimulus properties, and lighting.

### Eye movement recording and analysis

Zebrafish larvae at 5 dpf were tested. To prevent body movement without restricting eye movement, we embedded each larva dorsal side up in the center of a low-melting agarose drop (Sigma Type VII-A)^59^ on the bottom of a transparent glass plate. A platinum wire tool was used to remove the agarose around the head region so that the eyes could move freely in their regular medium, water. Thereafter, the larva-containing plate was completely submerged in the center of a translucent screen-wrapped glass cylinder filled with water and illuminated from below with infrared (IR)-emitting diodes (λ_peak_ = 875 ± 15 nm, OIS-150 880, OSA Opto Light GmbH, Germany). The movements of both eyes were recorded at a sampling rate of 40 frames per sec by an IR-sensitive charge-coupled device (CCD) camera. The area around the eyes was manually selected as the region of interest. For each larva, spontaneous eye movements in the dark or in the presence of illuminated stationary gratings were recorded for 5 min, followed by a counterclockwise-rotating optokinetic stimulus lasting for the scheduled duration (3, 4, 5, 6, 7, 10, 20, 30 or 40 min). Throughout this paper, both the horizontal eye rotation and the rotating optokinetic stimulus in the counterclockwise direction are marked as positive. After the stimulus ended, either the light was turned off or the gratings were turned to stationary, and the recording of the OKAN eye movement continued until the larva regained its spontaneous eye movements. Data were analyzed using custom-developed software written in MATLAB (MathWorks, Natick, MA, USA). Eye-position traces were smoothened using a Gaussian filter with a cutoff frequency of 5.5 Hz to increase the signal/noise ratio^44^. Eye movement velocity was calculated as the derivative of eye-position traces. To prevent the eye trace smoothing procedure from changing saccades into the slow phase data, we applied a three-step desaccading procedure to correctly identify quick- and slow-phases. Quick-phases and/or saccade eye movements were identified by a velocity threshold of 20 deg/sec that was applied to median velocities over a time window of every 0.5 sec and an eye dislocation threshold of 1 deg followed by manual adjustments^60,61^. Following desaccading the SPV was estimated as the median velocity in the first second of each slow phase. Similarly, we also computed the median eye position and used both data to draw the V-P (SPV versus Position) plot. The linear regression of velocity on position was estimated with ordinary least square by applying the fitlm function in MATLAB. We categorized the quick phases according to the velocity sign into positive and negative groups (Fig. 2A), followed by computing the QPF with counting the number of quick-phase eye movements in a time window of every 10 sec. To estimate the kinetics of the OKN adaptation, we fitted both the SPV and the change in quick-phase frequency (∆QPF), which was obtained by subtracting the positive QPF from the negative QPF, with a second-order exponential decay function:

1 $$f(t)=G1\cdot {e}^{-\frac{t-t0}{{\tau }_{1}}}+G2\cdot {e}^{-\frac{t-t0}{{\tau }_{2}}}+{\rm{offset}}$$

where f(t) represents SPV or ∆QPF function of time (t), *G1* is the gain of the first decay phase, *t*_0_ is the onset of optokinetic simulation, τ_1_ is the time constant of the first decay phase, *G2* is the gain of the second decay phase, and τ_2_ is the time constant of the second decay phase.

To estimate the decay kinetics of negative OKAN, we fitted both the SPV and ∆QPF with a first-order exponential decay function:

2 $$f(t)=G\cdot {e}^{-\frac{t-tc}{\tau }}$$

where f(t) represents SPV or ∆QPF function of time (t), *G* is the gain, *t*_*c*_ is the cessation of optokinetic simulation, τ is the time constant of decay and “offset” is not considered because SPV and ∆QPF are assumed to return to 0 gradually after the stimulation.

### Model

To better understand how the experimentally-observed set-point adaptation occurs, we used a nonlinear model with a set of adaptation and habituation leaky integrators (Fig. 8) that is described by different sets of equations during stimulus presentation and after stimulus offset (i.e., during darkness). During stimulation, the model is described by the following set of differential equations:

3 $$\begin{array}{ccc}\dot{X} & = & \frac{1}{1+gh}[\begin{array}{ccc}-\frac{1+g({k}_{a}+h)}{{T}_{a}} & \frac{{k}_{a}}{{T}_{a}} & -\,\frac{g{k}_{a}}{{T}_{a}}\\ -\frac{{k}_{VSM}}{{T}_{VSM}} & -\frac{1+h({k}_{VSM}+g)}{{T}_{VSM}} & \frac{{k}_{VSM}}{{T}_{VSM}}\\ \frac{g{k}_{h}}{{T}_{h}} & -\frac{{k}_{h}}{{T}_{h}} & -\frac{1+g({k}_{h}-h)}{{T}_{h}}\end{array}]X\\  &  & +\frac{1}{1+gh}[\begin{array}{c}\frac{gh{k}_{a}}{{T}_{a}}\\ \frac{{k}_{VSM}h}{{T}_{VSM}}\\ \frac{{k}_{h}}{{T}_{h}}\end{array}]{V}_{S},\end{array}$$

4 $${V}_{e}=\frac{1}{1+gh}[-g,1,-\,g]X+(\frac{gh}{1+gh}){V}_{s},$$

where $X={[A,Q,H]}^{T}$, *V*_*e*_ is the eye velocity, and *V*_*s*_ is the stimulus velocity. Here *A*, *Q* and *H* are the outputs of the leaky integrators of the adaption operator, velocity storage model and habituation operator, respectively. Moreover, *g* is the oculomotor gain; *k*_*VSM*_ and *T*_*VSM*_ are velocity storage gain and time-constant; *k*_*a*_ is adaptation gain, and *T*_*a*_ is adaptation time constant; *h* is habituation gain; *k*_*h*_ is habituation gain, and *T*_*h*_ is habituation time constant; *V*_*r*_ denotes the retinal slip velocity.

After stimulus offset (onset of the darkness), the model is described by the following set of differential equations:

5 $$\dot{Y}=[\begin{array}{cc}-\frac{1+g{k}_{a}}{{T}_{a}} & \,\frac{{k}_{a}}{{T}_{a}}\\ -\frac{{k}_{VSM}}{{T}_{VSM}} & -\frac{1}{{T}_{VSM}}\end{array}]Y,$$

6 $${V}_{e}=[-g,1]Y,$$

where $Y={[A,Q]}^{T}$ and *A* and *Q* are defined as previously. The model was simulated using Simulink in MATLAB (the Mathworks, Natick, MA). We used global optimization (*globalSearch* function in MATLAB) in order to maximize the variance accounted for (VAF) defined by:

7 $$VAF=1-\frac{var({V}_{e}-{V}_{e,measured})}{var({V}_{e,measured})},$$

where *V*_*e,est*_ and *V*_*e,measured*_ are the model estimated and experimentally measured values of eye velocity, respectively, and *var*(.) denotes the variance. We used nonlinear least squares method and trust-region-reflective algorithm to optimize the parameters of the model.

## Supplementary information

Supplementary information

## Data Availability

Data is available from the corresponding author upon reasonable request.

## Code availability

The custom analysis code used in this study is available from the corresponding author upon reasonable request.
