# Supplementary material for: Negative optokinetic afternystagmus in larval zebrafish demonstrates set-point adaptation
Source: Sci Rep. 2019 Dec 13;9:19039. doi: 10.1038/s41598-019-55457-4 (PMC6910917; doi:10.1038/s41598-019-55457-4)
Supplement: Supplementary file 1 — Supplementary information [file 41598_2019_55457_MOESM1_ESM.pdf]

Title: Negative optokinetic afternystagmus in larval zebrafish demonstrates set-point adaptation

Author list and affiliations: Ting-Feng Lin[1,2], Mohammad Mohammadi[3], Ahmed M. Fathalla[1,4], Duygu Pul[1], Dennis Lüthi[1], Fausto Romano[1], Dominik Straumann[1,2], Kathleen E. Cullen[5,6], Maurice J. Chacron[6], Melody Ying-Yu Huang[1,2]*

[1]*Department of Neurology, University Hospital Zurich, University of Zurich, Zurich, Switzerland*

[2]*Neuroscience Center Zurich (ZNZ), University of Zurich and ETH Zurich, Zurich, Switzerland*

[3]*Department of Biomedical Engineering, McGill University, Montreal, Quebec, Canada*

[4]*Current address: Monash Biomedicine Discovery Institute, Monash University, Melbourne, Australia.*

[5]*Department of Biomedical Engineering, The Johns Hopkins University, Baltimore, Maryland, USA*

[6]*Department of Physiology, McGill University, Montreal, Quebec, Canada*

*Corresponding author: Melody Ying-Yu Huang (melody.yingyu.huang@gmail.com).

Supplementary Information

Supplementary Figure S1

Poststimulatory eye movements of negative OKAN and the influences of velocity storage. **A-B.** Acceleration (build-up phase) of negative OKAN in single zebrafish larvae recorded during the first 2 min in darkness after 10 (**A**) and 40 min (**B**) of OKN. **C.** A silent period before the negative OKAN emerges recorded during the first 2 min in darkness after 7 min of OKN in a larva. In all recordings the optokinetic stimulus velocity was 10 deg/sec.

Supplementary Figure S2

The relationship between SPV and ΔQPF. The average SPV was plotted against the average ΔQPF of 15 fish. Each data point represents 10 sec of the total 45-min recording. The data collected from the left eye and right eye are shown as blue and red lines, respectively. Circles indicate prestimulatory data, squares indicate stimulatory data and diamonds indicate poststimulatory data.

Supplementary Figure S3

Conceptual models and simulation results of sensory habituation and set-point adaptation. Conceptual models of sensory habituation alone (**A**) and the simulated SPVs under 10-min optokinetic stimulation of 10 deg/sec (**B**). Conceptual models of set-point adaptation alone (**C**) and the simulated SPVs under 10-min optokinetic stimulation of 10 deg/sec (**D**). (**A** and **C**) The black part represents the optokinetic negative feedback control of the retinal slip velocity incorporating the velocity storage mechanism. The blue part describes the sensory habituation, which is composed of one gain and one leaky integrator (**A**). Sensory habituation adjusts how efficiently the initial sensory input can be converted into electrochemical signals. T_h is time constant, while the reciprocal of k_h indicates how leaky the integrators is. The red part describes the set-point adaptation (**C**). Adaptation operator is composed of a leaky velocity integrator. T_a is time constant, while the reciprocal of k_a indicates how leaky the integrator is. (**B** and **D**) The data collected from the right eye is shown as red lines (see Fig. 5A and 5B).

The simulated SPVs under 10-min optokinetic stimulation of 10 deg/sec is depicted as black lines. The optimized parameters are shown in Supplementary Table S5.

Supplementary Table S1. Pearson correlation coefficients and the corresponding p-values between SPV and ΔQPF shown in Supplementary Figure S2. Data are solicited from 15 fish.

Supplementary Table S2. Estimated negative OKAN decay time constants±95% confidence interval (min). SD = stimulus duration (min).

Supplementary Table S3. Estimates±standard deviation of regression lines shown in Figure 6. Two-tailed t-test was used to compare the estimated y-intercepts and slopes between pre- and poststimulations. n = sample numbers.

Supplementary Table S4. Parameters of simulations shown in Figure 10. SD = stimulus duration (min). *VAF* = variance accounted for.

Supplementary Table S5. Parameters of simulations shown in Figure 11. SD = stimulus duration (min).

Supplementary Figure S1

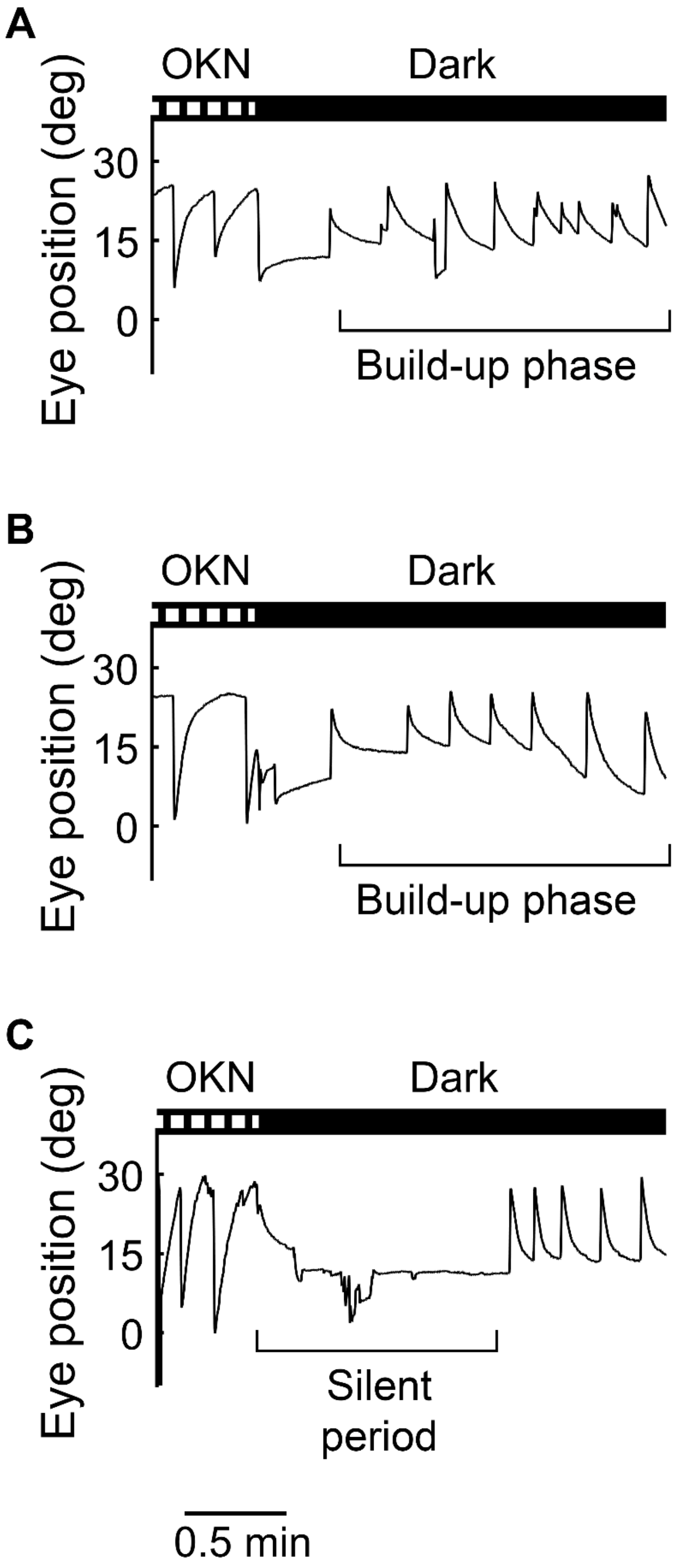

Supplementary Figure S2

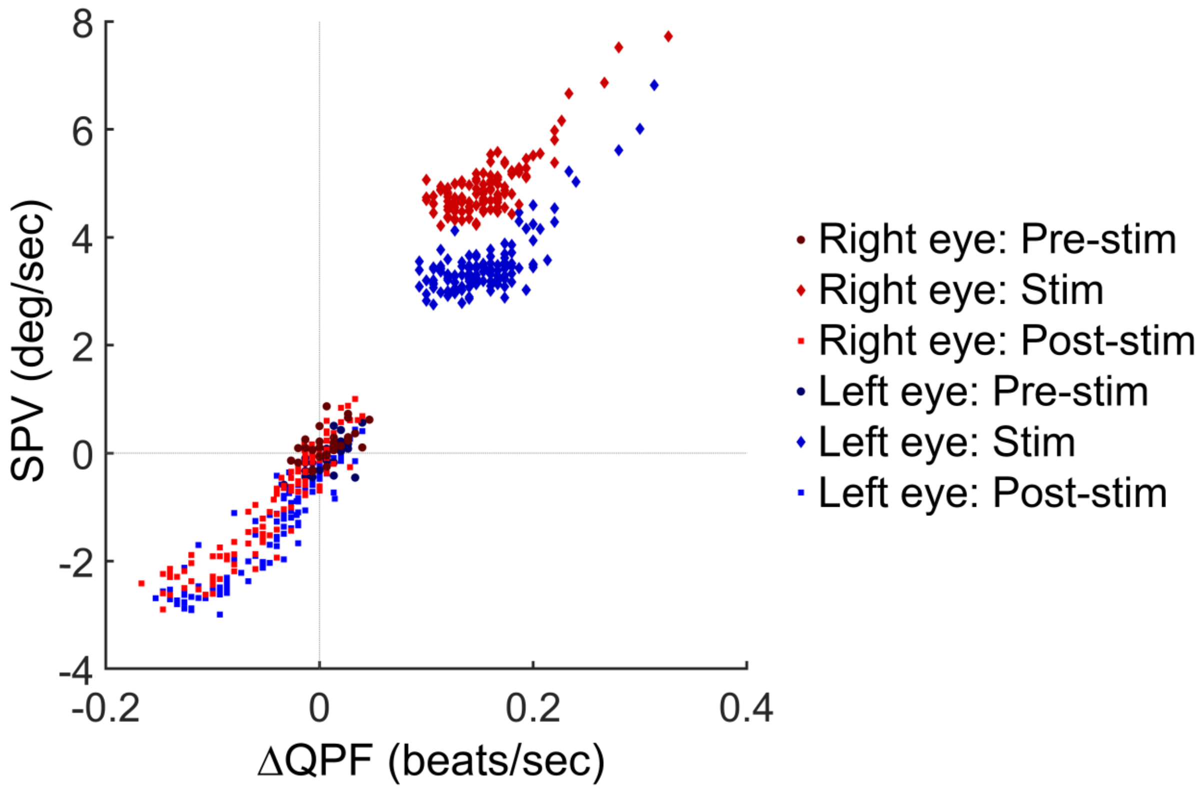

Supplementary Figure S3

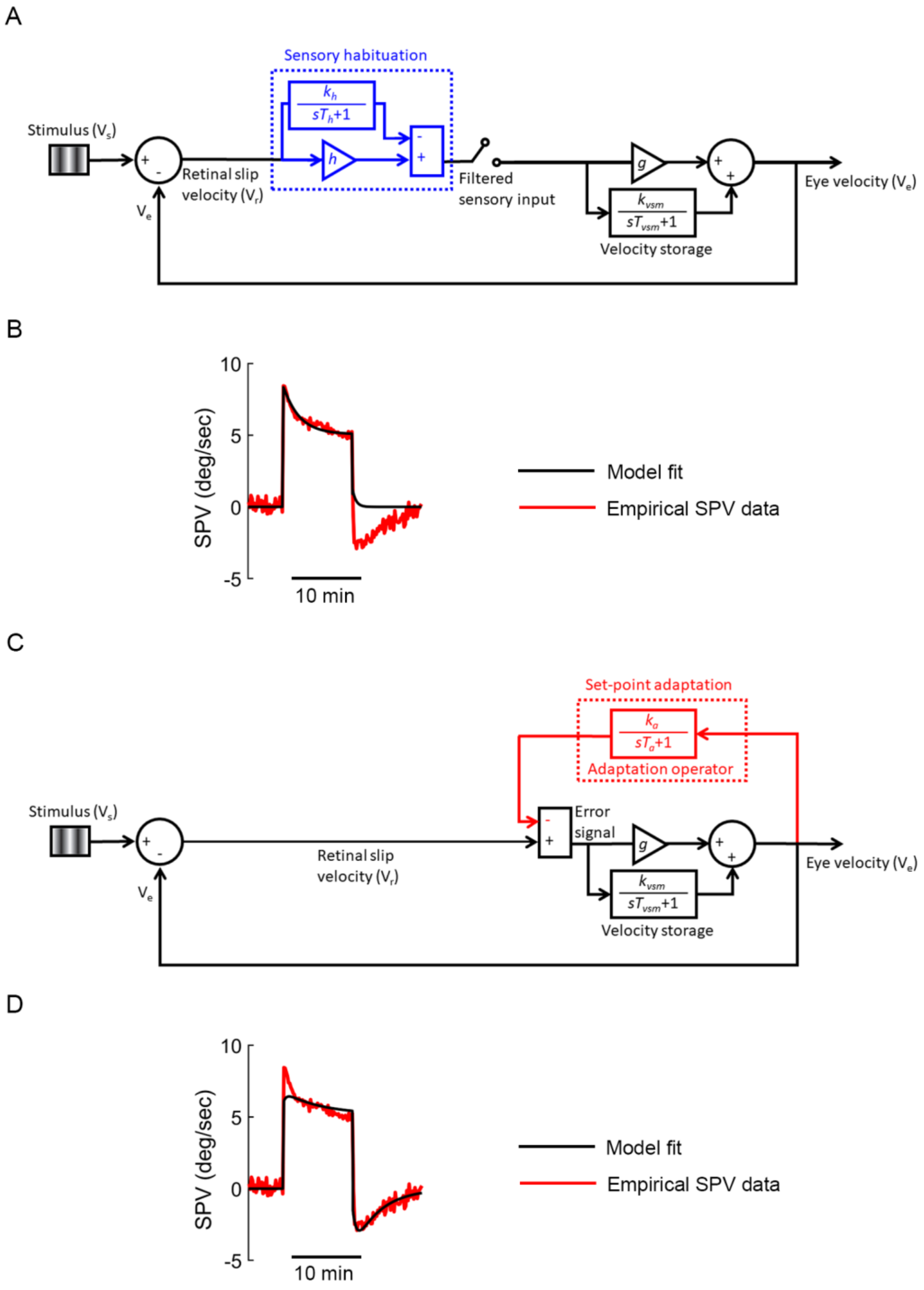

Supplementary Table S1

	Left eye		Right eye	
	Pearson r	P-value	Pearson r	P-value
Whole recording (n=270)	0.98	3.806e-183	0.98	1.952e-178
Prestimulation (n=30)	0.63	1.838e-04	0.53	0.003
Stimulation (n=120)	0.77	1.848e-24	0.80	2.142e-28
Poststimulatino (n=120)	0.92	3.534e-51	0.94	5.911e-55

Supplementary Table S2

	Left eye		Right eye	
SD	SPV	∆QPF	SPV	∆QPF
5 (n=23)	5.37±1.62	3.18±1.08	1.84±0.65	2.74±0.90
6 (n=17)	5.05±1.35	3.04±1.05	2.93±0.82	3.09±0.94
7 (n=21)	6.20±1.20	4.27±1.03	4.78±0.10	4.80±1.18
10 (n=23)	3.91±0.72	3.52±0.70	3.95±0.81	3.41±0.75
20 (n=15)	7.03±0.79	4.22±0.60	5.02±0.81	3.94±0.55
40 (n=8)	9.14±0.99	7.83±1.20	8.62±1.14	7.80±1.15

Supplementary Table S3

	Left eye		Right eye	
	Y-intercept	Slope	Y-intercept	Slop
Prestimulation	-1.210±1.884<br><br>(n=505)	-0.151±0.144<br><br>(n=505)	1.252±2.295<br><br>(n=477)	-0.136±0.172<br><br>(n=477)
Poststimulation	-2.878±1.474<br><br>(n=584)	-0.122±0.277<br><br>(n=584)	-1.773±4.906<br><br>(n=584)	-0.078±0.274<br><br>(n=584)
P-value	1.632e-53	0.030	3.768e-63	3.044e-05

Supplementary Table S4

	Left eye						Right eye					
SD	5	6	7	10	20	40	5	6	7	10	20	40
T_a	1300	1300	1300	1300	1300	1300	700	700	700	700	700	700
T_{vsm}	30	30	30	30	30	30	30	30	30	30	30	30
T_h	10	10	10	10	10	10	10	10	10	10	10	10
g	0.9	0.7	0.9	0.9	0.5	0.9	1.6	0.9	1.7	1.5	0.7	1.3
k_{vsm}	0.5	0.8	0.5	0.7	0.7	0.5	0.1	0.6	0.7	0.55	0.3	1.2
k_a	2.2	2	1.8	1.4	1.6	1.4	0.5	1.1	0.45	0.45	0.85	0.4
h	3.5	4	4.5	5	5	5.1	9	7.2	7.2	7	7	8
k_h	2.9	3.5	3.9	4.35	4.25	4.2	8	6.3	6.4	6.2	5.6	7.2
VAF	0.98	0.97	0.98	0.98	0.97	0.96	0.98	0.98	0.99	0.99	0.98	0.97

Supplementary Table S5

	Right eye	
	Habituation	Set-point adaptation
T_a		438
T_{vsm}	30	30
T_h	32	
g	1.5	1.5
k_{vsm}	0.55	0.55
k_a		0.45
h	3.6	
k_h	3.1	
